# Celluloid

**Published:** 1878-02

**Authors:** J. B. Tullis


### CELLULOID.

BY J. B. TULLIS, D. D. S.

In consequence of the royalty of the rubber company demanded of the dentist, other materials have been introduced as a cheap base for artificial teeth.

Celluloid has been more generally used than any other of which we know. Like every thing new it has had its strong friends, its successes and failures. I began its use in January, 1872. The first plates, or many of them were defective, and consequently did not do well. The improved are much better. The mode of working has much to do with the final success. I will give a brief outline of the way I have used celluloid, as it may aid some of the younger members of the profession in its use. First get a correct impression of the mouth, use your trial plate, grind and fit your teeth and wax up, as for rubber base. Let your plaster be stiff for investing the teeth. Reverse the flask when you put it in the bath. The cutting edges of the teeth will all be down, and then there will be no moving the teeth from their proper places.

Put the plate on top, and bring the flask together slightly, so as not to break the edges of the plaster model. Cut the waste gates in the upper model, and, as you close the flask, press the material down around the teeth, and the excess is forced up into the waste gates. Turn down slowly until the flask comes together, then press very hard, and let it remain in the oil or glycerine—glycerine is more used now I think, than oil, and is more cleanly.

Let it remain about thirty minutes, and then let it cool in the bath, and remain in press at least six hours or all night. The material is then fully set, and not so easily sprung in dressing up; leave the wax a little full over the pins of the teeth, and when you dress up, you will not then expose them. If you wish an air chamber you can cut a piece of lead, the size and thickness you desire, and confine it in place by a pin in the center. Finish up with scrapers, and powder as you do rubber.

A little patience and experience will enable you to produce good results.

